# Placenta response of inflammation and oxidative stress in low-risk term childbirth: the implication of delivery mode

**DOI:** 10.1186/s12884-017-1589-9

**Published:** 2017-12-06

**Authors:** Yabin Hu, Kun Huang, Yuanfang Sun, Jianqing Wang, Yeqing Xu, Shuangqin Yan, Peng Zhu, Fangbiao Tao

**Affiliations:** 1 Department of Maternal, Child and Adolescent Health, School of Public Health, Anhui Medical University, No. 81 Meishan Road, Hefei, 230032 Anhui People’s Republic of China; 2 Ma’anshan Maternal and Child Health (MCH) Center, Ma’anshan, Anhui People’s Republic of China; 3 Anhui Provincial Key Laboratory of Population Health & Aristogenics, Hefei, Anhui People’s Republic of China

**Keywords:** Delivery mode, Placenta, Inflammation, Oxidative stress

## Abstract

**Background:**

Caesarean delivery rate is increasing gradually in China and there is no doubt that delivery mode is closely associated with the maternal health and infant development.This study examined the independent effect of delivery mode on placenta inflammation response and oxidative stress response.

**Methods:**

A total of 3474 pregnant women recruited in Ma’anshan Birth Cohort Study were the initial study population. Data on maternal socio-demographic characteristics and pre-pregnancy BMI were collected at their 1st antenatal checkups. Pregnancy-specific anxiety was assessed during the three trimesters of pregnancy. Common pregnant complications were monitored in the whole pregnancy period. Delivery modes, as well as newborn characteristics were abstracted from medical records. Delivery modes included vaginal deliveries (VD), caesarean delivery with medical indications (CDMI), caesarean delivery on maternal request (CDMR) and urgent cesarean delivery (UCD). Placentas were collected during childbirth. The mRNA expression of IL-1β, TNF-a, IL-6, IFN-γ, IL-4, IL-10, IL-8, and HO-1 were assessed in the final sample of 1978 low-risk women with singleton term-births.

**Results:**

The overall rate of caesarean delivery (CD) was 50.5% (1650/3265) in singleton term childbirths in this study. Among women who reported definite CD reasons, 56.8%of them chose the surgery without any medical indications.It shows a non-linear relationship between cytokines related with placenta inflammatory response, oxidative stress response and different delivery modes. At high percentiles of IL-1β, IFN-γ and IL-8, women with CDMR had higher expression levels compared to women with VD. Women with CDMI had higher levels at median percentiles of IL-1β, IFN-γ and IL-8. Women with CDMR had higher expression compared with VD at high percentiles of IL-6 and HO-1, and women with CDMI had higher levels of these two cytokines at their low percentiles. It is worth noting that at high percentiles, compared with normal delivery, the expression of IL-1β, IFN-γ, IL-8 and HO-1 have significantly altered in women with CDMR.

**Conclusion:**

A high overall CD rate was found in this study, and caesarean delivery on maternal request was the major contributor to the high prevalence. Maternal placental oxidative stress and inflammatory response were closely associated with delivery mode. The effect is much amplified at high levels of expression in women who chose CD on maternal request.Such difference needs to be noticed and may have important implications for obstetricians, midwives and other perinatal health care workers.

## Background

Caesarean delivery (CD) is a surgical procedure performed when a vaginal delivery (VD) would put the baby or mother at risk. In the past decades, the incidence of CD has dramatically risen worldwide. In the United States, the CD rate has increased from 5.8% in 1970 to 32.3% in 2008 [1]. Similarly, it has been increasing rapidly from 1980s in developing countries. As showed in the WHO report, the overall rate of CD was 27.3% in Asia in 2007–2008, and China had the highest rate of 46.2% among them and 40% of cesareans were reported to have no clinical indications [2]. Recently, WHO Global Survey of Maternal and Perinatal Health (WHOGS; 2004–08) and the WHO Multi-Country Survey of Maternal and Newborn Health (WHOMCS; 2010–11) reported that the CD rate of China has already increased to 47.6% [3]. Based on the latest data from 150 countries in Asia, arecent study suggested that the CD rate in Wuhan of China was over 50% [4]. It’s worth noting that elective caesarean delivery (ECD) contributes mainly to this uprising trend [5, 6]. ECD is commonly arranged ahead of time before labor, including the planned surgery due to medical indications (CD with medical indications, CDMI) which have developed before or during the pregnancy, and scheduled operation defined as on maternal request without medical indications (CD on maternal request, CDMR). CDMR was the predominant contributor to the increase of CD in China [5, 7–9].

As a strong acute process of stress, childbirth can cause a lot of endocrine and immunological change both in women and in neonates. Both inflammation and oxidative stress responses are especially remarkable during this period. Inflammatory and oxidative stress responses act as crucial part in immune functions and neural development [10, 11]. Oxidative stress has been suggested as a causative agent in human pregnancy-related disorders, such as embryonic resorption, recurrent pregnancy loss, preeclampsia, intra-uterine growth restriction, and fetal death [12]. It is worth noting that the labor plays an important role in the expression of inflammation response and/or oxidative stress response. Myometrium contractility and cervix dilation stimulated by the elevating level of prostaglandin biosynthesis in vaginal delivery are accompanied with high fluctuation of cytokines, such as interleukin-1β (IL-1β), IL-6 and tumor necrosis factor-a (TNF-a) [13]. It is now well-known that oxidative stress increases during normal pregnancy, and woman will experience different degrees of oxidative stress in cesarean delivery and vaginal delivery [14, 15]. Hung et al. [15] showed evidence of increased placental oxidative stress in 37 women with vaginal deliveries as compared with other 36 women with elective cesarean sections. Bakheitet al [16], using a sample of 76 women, had found concentrations of interferon γ (IFN-γ), IL-4 and IL-10 in the peripheral and placental sera were higher in VD compared to ECD.

Previous studies that focused on the association of delivery mode with inflammation response or oxidative stress response have mainly used a cross-sectional or register-based study design. As is known that experiences and exposures during pregnancy will distinctly alter maternal and placenta inflammation and oxidative stressresponses, such as maternal psychological stress and severe pregnant complications [17–19]. Jun et al. [17] showed more macrophages accumulate in placenta ofpregnancy complicated with gestational diabetes mellitus (GDM), and the expression levels of pro-inflammation factors are also increasedin GDM pregnancies, suggesting that macrophages and inflammatory mediators (IL-6 andTNF-α) may play an important role in GDM. The fetus of diabetic mothers develops inan inflammatory milieu. Radaelliet al [18] speculated that changes inexpression of specific placental genes may be a leadingcause to adverse fetal programming in GDM pregnancies. Bronson et al. [19] demonstrated that maternalstress induces placental inflammation, the levels of pro-inflammatory cytokines IL-6 and IL-1β increased, specifically inmale placentas. However, cross-sectional study design cannot address the causal relationship between delivery mode and inflammation response or oxidative stress response. Thus, based on a large community-based birth cohort, this study aimed to investigate the independent effect of delivery mode on the placenta inflammation and oxidative stress responsesin low-risk term childbirths.

## Methods

### Participant recruitment

Participants of this study were recruited into the Ma’anshan Birth Cohort Study (MBCS), which was conducted in the maternal and child health center in Ma’anshan city, Anhui Province, China. Women were invited to participate in the MBCS at the 1st antenatal visit (before 14 gestational weeks) by special maternal and childcare doctors from May 2013 to September 2014. All participants provided written informed consents after understanding the aim and content of this study. The study was approved by the Ethical Committee of Anhui Medical University (number: 20,131,401).

### Maternal and newborn characteristics

#### Socio-demographic characteristics

Baseline socio-demographic data were collected from all participated women for the subsequent follow-ups. Women were asked to fill in a questionnaire in each trimester of pregnancy. General socio-demographic characteristics were collected in the 1st trimester’s questionnaires, including maternal age, household income, gravidity and previous adverse pregnant outcome. Body height and body weight were measured during the 1st antenatal visit. The body weight was regarded as pre-pregnancy body weight and pre-pregnancy body mass index (BMI = kg/m^2^) was calculated from weight (kg) divided by the square of height (m^2^).

#### Pregnancy-specific anxiety assessment

In each trimester of pregnancy, pregnancy-specific anxiety was assessed by a pregnancy-specific anxiety questionnaire developed by our research team [20]. It is a 4-point Likertquestionnaire consisting of 13 items. It covered three dimensions: anxiety regarding women’s own health, anxiety on fetal growth and development and anxiety about the safety and success of childbirth process. Women were asked to self-rate their perception from 1 to 4 points varying from no worries, occasionally worried, often worried to always worried. Scores ranged from 13 to 52, higher scores indicating higher level of pregnancy-specific anxiety. The test–retest reliability coefficient and Cronbach’s alpha coefficient were 0.79 and 0.81, respectively. Confirmatory factor analysis showed that the values of root mean square error of approximation (RMSEA), goodness-of-fit index (GFI), normed fit index (NFI) and comparative-fit index (CFI) were 0.07, 0.95, 0.90 and 0.91, respectively. It is the first instrument to assess pregnancy-specific anxiety and maternal life events in China and is regarded as an appropriate tool for maternal psychosocial evaluation and intervention [20].

#### Monitoring of pregnant complications

Blood pressure, blood sugar and total bile acid (TBA) levels were continuously monitored in the entire pregnancy. Hypertension disorders complicating pregnancy (HDCP) includes pregnancy-induced hypertension, preeclampsia or eclampsia, pregnancy with chronic hypertension, and chronic hypertension complicated with preeclampsia. The diagnostic criteria for gestational hypertension: 1. blood pressure was firstly found to rise in pregnancy (systolic blood pressure ≥ 140 mmHg or diastolic blood pressure ≥ 90 mmHg); 2. no proteinuria; 3. blood pressure decreased to normal level within 12 weeks after delivery. Preeclampsia was diagnosed as: systolic blood pressure ≥ 140 mmHg or diastolic blood pressure ≥ 90 mmHg, together with proteinuria ≥0.3 g/24 h or random urine protein (+), might complicate with upper abdominal discomfort, headache and other symptoms in women after 20 gestational weeks.

GDM was screened on week 24–28 of pregnancy. In the medical records, GDM was confirmed by the standard diagnosis protocol in China using a 75 g oral glucose tolerance test (OGTT). Women were asked to have an oral glucose after fasting for 12 h. Fasting level of blood glucose, blood glucose level of 1 h and 2 h after oral administration were assessed. The threshold was set as: (i) fasting blood glucose ≥5.6 mmol/L, (ii) blood glucose level at 1 h after oral administration ≥10.3 mmol/L, (iii) blood glucose level at 2 h after oral administration ≥8.6 mmol/L. GDM was diagnosed when no less than two of the three parameters reached or exceeded the normal value. Women with existing pregestational diabetes mellitus were excluded from the sample.

Intrahepatic cholestasis of pregnancy (ICP) was diagnosed when TBA level ≥ 10 μmol/L.

#### Collection of newborn characteristics data

Data on childbirth, such as gestational age at birth, infant gender, birth weight, 1-Min Apgar score, 5-Min Apgar score and placental size (mm^3^ = Length*Width*Height) were abstracted from medical records. Infants with gestational age under 37 weeks were defined as preterm infants. Delivery modes included vaginal deliveries (VD), CDMI, CDMR and urgent cesarean delivery (UCD) in this study. CDMR was defined as CD due to maternal request with no medical indicationsinterm single birth. CDMI, together with CDMR, were defined as elective CD (ECD). UCD was defined as an urgent CD conducted due to medical indications or other causes after the onset of labor.

### Measurement of inflammation and oxidative stress responses

#### Collection of placenta tissues

Placenta tissues were collected within 30 min after delivery. Washed by normal saline, one single lobule was taken out in the placenta vertically where there was no calcification or fascia. It was then divided into 4 average parts and fixed quickly in liquid nitrogen. A total of 2554 placental specimens were assessed in this study.

#### qRTPCR measurement of inflammatory cytokines and oxidative stress cytokines

Pro-inflammatory cytokines (including IL-1β, IL-6, IFN-γ and TNF-α), anti-inflammatory cytokines (like IL-4, IL-10), and pro-inflammatory chemokines (IL-8) were selected according to the literature [10, 21–26]. Heme-oxygenase-1 (HO-1) was regarded as biomarker of oxidative stress. All RNA quality was assessed using Nanodrop® ND-1000 (Nano Drop, USA), then total RNA (1.0 μg) was reverse-transcribed into cDNA using the AMV Reverse Transcription System (Promega, USA) according to the manufacturer’s instructions with the value of 260/280 ≥ 1.8. Quantitative real-time polymerase chain reaction was used to measure the mRNA expression of each cytokine. The amplification reactions were runon a Light Cycler® 480II Instrument (Roche, Germany). In addition, the real-time PCR running protocol was an initial hold step (95 °C for 10 min) and 45 cycles of a three-step PCR (95 °C for 15 s, 60 °C for 15 s, 72 °C for 20 s). All RT-qPCR data were normalized through an endogenous reference RNA, 18SrRNA. Delta Ct (ΔCt) was defined as the expression difference between the target mRNA and the normalizing RNA: ΔCt = Ct mRNA- Ct normalizing RNA.Due to the large sample size, we could not measure the gene concentration duplicate. In order to certify the quality of measurement, we tripled measured all cytokines randomly using 4 samples in qPCR, and the coefficients of variation were almost less than 1.

### Statistical analysis

To reach the normality and the homogeneity of the variance assumptions, our mRNA expression values were transformed to the natural logarithm. One-way ANOVA was used to evaluate maternal factors, infants’ characteristics and maternal placental mRNA expression of cytokines in 4 kinds of delivery modes. Pairwise comparisons were performed using LSD test. Pearson Chi-Square Test (χ^2^) was used to assess the rate of infant gender and rate of previous adverse pregnant outcomes among the 4groups. Quantile regression model was utilized to adjust for confounding factors, including pre-pregnancy BMI, maternal age, gestational age, gravidity, previous adverse pregnant outcome, pregnancy-specific Anxiety in 1^st^, 2nd and 3rd trimesters, infant gender, birth weightand placental size.Statistical tests were two-sided and performed with stata 14.0. All results were considered statistically significant at *P* value <0.05.

## Result

### Rate of caesarean delivery

**Fig. 1:**
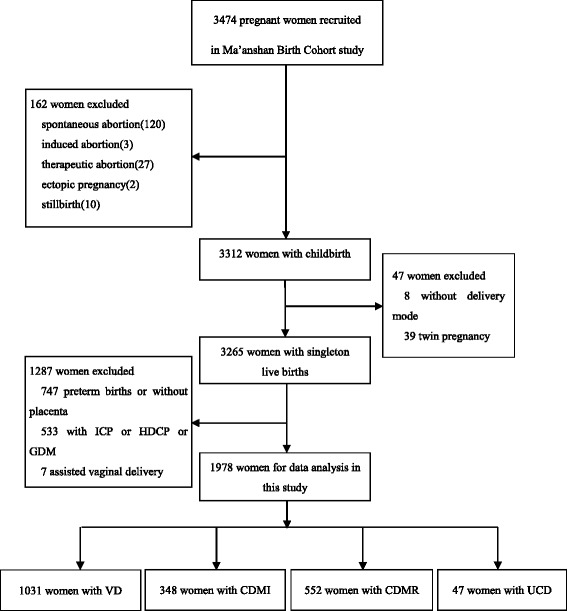
Participants recruitment flow chart. *ICP* intrahepatic cholestasis of pregnancy; *HDCP* hypertension disorders complicating pregnancy; *GDM* gestational diabetes mellitus; *VD* vaginal delivery; *CDMI* cesarean delivery with medical indication; *CDMR* cesarean delivery on maternal request; *UCD* urgent cesarean delivery

As showed in Fig. 1, in a total of 3474 women, 162 women were excluded due to different kinds of abortion, ectopic pregnancy and stillbirth. There were 3312 women with childbirth in the cohort study. Eight women without delivery mode records and 39 with twin pregnancies were further excluded. Thus, the overall rate of CD was 50.5% (1650/3265) in singleton live births in this study. Due to 112 missing data on reasons for CD, totally 1538 women choosing CD had definite causes. Among those women, 94.9% (1459/1538) chose ECD, in which CDMR and CDMIaccounted for 56.8% (828/1459) and 43.2% (631/1459), respectively.

### General characteristics of participants

According to the study design, women with no information on delivery mode, twin pregnancies, women without placenta,and women with preterm birth and pregnancy complications such as ICP or HDCP or GDM were excluded. We also excluded a small sample of 7 assisted vaginal deliveries. Finally, 1031 women with VD, 348 with CDMI, 552 with CDMRand 47 with UCD were included as the study sample for data analysis (Fig. 1).

We found that maternal age, gravidity and pre-pregnancy BMI of participants included were lower than those excluded (P <0.05). Gestational age of included women was higher compared to those excluded (*P* <0.001). Newborns’ height, 1-Min Apgar score and 5-Min Apgar score of included infants were higher than those excluded (*P* <0.05).

The included participants’ characteristics were summarized in Table 1. Significant differences in maternal age, gravidity, pre-pregnancy BMI, previous adverse pregnant outcomes, birth weight, birth height, head circumference and placental size among 4 groups were observed. Women who chose VD were younger than those who chose CD (P<0.001), and maternal age was highest in women choosing CDMI. The gravidity was lower in women who terminated pregnancy with VD than those with ECD (P<0.001), in which women with CDMI had higher gravidity than women with CDMR (*P* = 0.002). Women with UCD had the highest pre-pregnancy BMI while those with VD had the lowest value (*P*<0.001). No significant differences were found in pregnancy-specific anxiety scores across the three trimesters of pregnancy. Newborns from CD had higher birth weight than those from VD (P<0.001), those from UCD being the highest. Infants born by ECD had higher head circumferences than those born by VD (P<0.001). CDMI infants had the highest head circumference among the four groups. As to the placental size, women choosing VD had smaller sizes than those who chose CDMI (*P*<0.001), CDMR (*P*<0.001) or UCD (*P* = 0.038).

**Table 1:** Maternal and infants’ characteristics in 4 kinds of delivery modes

Characteristics	VD	CDMI	CDMR	UCD	*P* Value
Maternal characteristics					
Maternal age(years)	25.95 ± 3.02	27.41 ± 3.83	26.48 ± 3.73	27.15 ± 2.87	<0.001
Gestational age(weeks)	39.20 ± 1.22	39.12 ± 1.37	39.29 ± 1.06	39.15 ± 1.18	0.191
Gravidity	1.55 ± 0.80	1.83 ± 1.04	1.65 ± 0.86	1.72 ± 0.83	<0.001
Pre-pregnancy BMI(kg/m^2^)	19.96 ± 2.32	20.73 ± 2.75	20.44 ± 2.66	20.88 ± 2.71	<0.001
Previous adverse pregnant outcome	638(61.9%)	187(54.0%)	317(57.7%)	24(51.1%)	0.032
Pregnancy-specific anxiety score in 1st trimester	20.26 ± 4.82	19.80 ± 4.26	20.55 ± 5.13	19.55 ± 3.72	0.191
Pregnancy-specific anxiety score in 2nd trimester	19.61 ± 4.44	19.91 ± 4.62	19.92 ± 4.90	19.27 ± 3.84	0.459
Pregnancy-specific anxiety score in 3rd trimester Infants’ characteristics	18.72 ± 4.00	18.96 ± 4.30	19.17 ± 4.63	18.76 ± 3.43	0.245
Gender					0.174
Male	544(52.8%)	163(46.8%)	281(50.9%)	28(59.6%)	
Female	487(47.2%)	185(53.2%)	271(49.1%)	19(40.4%)	
Birth weight(g)	3304.81 ± 379.63	3443.95 ± 467.26	3417.05 ± 397.17	3481.70 ± 350.30	<0.001
Birth height(cm)	50.13 ± 1.71	50.03 ± 1.76	49.95 ± 1.51	50.70 ± 1.54	0.013
Head circumference(cm)	33.82 ± 1.30	34.33 ± 1.73	34.20 ± 1.46	34.14 ± 2.04	<0.001
Placental size(cm^3^)	685.79 ± 180.12	815.70 ± 224.56	799.87 ± 207.96	754.31 ± 230.07	<0.001
Apgar score					
1-Min	9.95 ± 0.46	9.94 ± 0.53	9.97 ± 0.42	9.91 ± 0.46	0.784
5-Min	9.98 ± 0.23	9.99 ± 0.16	10.00 ± 0.04	10.00 ± 0.00	0.219

*VD* vaginal delivery, *CDMI* cesarean delivery with medical indication, *CDMR* cesarean deliveryon maternal request, *UCD* urgent cesarean delivery, *BMI* body mass index; data presented with mean ± SD and n(%)

### Distribution of cytokines related to inflammation and oxidative stress among various modes of delivery

As it was depicted for average expression level of cytokines related to inflammation and oxidative stress among various modes of delivery in Table 2, significant differences were found among VD, CDMI, CDMR and UCD groups in IL-1β, TNF-α, IL-8 and HO-1 mRNA expressions. In pro-inflammatory cytokines, the levels of mRNA expression were the highest in UCD women. Level of IL-1β mRNA expression was significantly higher in women with VD than those with CDMR (*P* = 0.007). TNF-α expressed lower in VD compared to those in CDMR or UCD (*P* = 0.003 and *P* = 0.031). In IL-8, level of mRNA expression either in CDMI or CDMR was significantly less than in VD(*P* = 0.009 and *P* <0.001), and it significantly increased in UCD women compared to those with CDMR(*P* = 0.049). About HO-1 mRNA expression, women with CDMI or with CDMR had significant higher level than those with VD (*P* = 0.039 and *P* = 0.002).

**Table 2:** Maternal placental mRNA expression of cytokines in 4 kinds of delivery modes (mean ± SE)

Cytokines	VD	CDMI	CDMR	UCD	*P* Value
Pro-inflammatory cytokines					
IL-1β	0.98 ± 0.04	0.85 ± 0.07	0.77 ± 0.06	1.04 ± 0.22	0.042
TNF-α	1.36 ± 0.05	1.47 ± 0.08	1.59 ± 0.06	1.84 ± 0.23	0.007
IL-6	0.73 ± 0.04	0.85 ± 0.06	0.90 ± 0.06	1.02 ± 0.22	0.063
IFN-γ	1.18 ± 0.06	1.12 ± 0.09	1.15 ± 0.07	1.59 ± 0.25	0.399
IL-8	0.87 ± 0.04	0.65 ± 0.07	0.55 ± 0.06	0.96 ± 0.20	<0.001
Anti-inflammatory cytokines					
IL-4	0.93 ± 0.05	0.81 ± 0.08	0.89 ± 0.06	1.19 ± 0.22	0.354
IL-10	1.07 ± 0.05	0.99 ± 0.08	0.96 ± 0.07	1.11 ± 0.23	0.538
Oxidative stress cytokines					
HO-1	0.91 ± 0.04	1.09 ± 0.08	1.15 ± 0.06	0.90 ± 0.16	0.009

*VD* vaginal delivery, *CDMI* cesarean delivery with medical indication, *CDMR* cesarean deliveryon maternal request, *UCD* urgent cesarean delivery

### Effect of delivery mode on cytokines related to inflammation and oxidative stress

Table 3 presented the adjusted effect of delivery modes on quintiles of IL-1β, TNF-α, IL-6, IFN-γ, IL-8, IL-10 and HO-1 mRNA expression in maternal placenta. The mode of delivery was the major independent variable and VD was regarded as the control group in the quintile regression model. The covariates that were adjusted for included pre-pregnancy BMI, maternal age, gestational age, gravidity, previous adverse pregnant outcome, pregnancy-specific anxiety scores in three trimesters of pregnancy, infant gender, birth weight and placental size.

**Table 3:** Adjusted effect of delivery modes on quintiles of IL-1β, TNF-α, IL-6, IFN-γ, IL-8, IL-10 and HO-1 mRNA expression of maternal placenta

Cytokines	Groups	Quantile regressions				
		10th	25th	50th	75th	90th
IL-1β	CDMI	0.11(−0.28,0.51)	0.03(−0.21,0.26)	−0.26(−0.51,-0.01)*	−0.20(−0.49,0.09)	−0.23(−0.59,0.13)
	CDMR	0.09(−0.25,0.43)	−0.14(−0.38,0.10)	−0.23(−0.50,0.03)	−0.30(−0.43,-0.17)*	−0.45(−0.67,-0.22)*
	UCD	0.43(−0.35,1.21)	0.39(−0.14,0.92)	−0.07(−0.57,0.44)	−0.01(−0.58,0.58)	−0.41(−1.53,0.71)
TNF-α	CDMI	0.20(−0.21,0.62)	0.15(−0.19,0.49)	0.12(−0.11,0.34)	−0.01(−0.30,0.28)	−0.02(−0.47,0.44)
	CDMR	0.31(−0.04,0.67)	0.27(−0.06,0.60)	0.20(−0.02,0.41)	0.03(−0.21,0.27)	0.06(−0.19,0.31)
	UCD	0.34(−0.51,1.19)	0.36(−0.19,0.90)	−0.02(−0.83,0.79)	−0.06(−1.36,1.24)	1.11(−0.60,2.83)
IL-6	CDMI	0.56(0.30,0.81)*	0.04(−0.12,0.20)	0.11(−0.07,0.28)	−0.06(−0.30,0.19)	0.06(−0.25,0.38)
	CDMR	0.39(0.55,0.73)*	0.15(−0.03,0.33)	0.19(−0.03,0.41)	0.27(0.09,0.45)*	0.20(0.06,0.35)*
	UCD	0.37(−0.50,1.25)	0.26(−0.10,0.62)	0.58(−0.41,0.53)	−0.31(−0.92,0.29)	0.64(−1.38,2.65)
IFN-γ	CDMI	0.20(−0.31,0.71)	0.12(−0.32,0.57)	−0.15(−0.46,0.17)	−0.14(−0.40,0.11)	−0.20(−0.69,0.28)
	CDMR	0.12(−0.25,0.48)	0.17(−0.39,0.72)	−0.07(−0.35,0.20)	−0.25(−0.48,-0.02)*	−0.23(−0.62,0.15)
	UCD	−0.13(−2.21,1.94)	0.32(−0.45,1.10)	0.05(−1.15,1.26)	0.33(−0.65,1.31)	0.26(−0.45,0.97)
IL-8	CDMI	0.03(−0.21,0.28)	−0.17(−0.48,0.14)	−0.21(−0.53,0.11)	−0.32(−0.63,-0.01)*	−0.37(−0.62,-0.13)*
	CDMR	−0.07((−0.30,0.16)	−0.34(−0.56,-0.11)*	−0.40(−0.64,-0.16)*	−0.40(−0.63,-0.17)*	−0.29(−0.53,-0.06)*
	UCD	0.15(−0.36,0.67)	−0.27(−0.90,0.36)	−0.01(−0.71,0.70)	0.10(−0.96,1.16)	−0.19(−1.25,0.86)
IL-10	CDMI	−0.06(−0.34,0.23)	−0.02(−0.31,0.27)	0.01(−0.34,0.35)	0.03(−0.44,0.49)	0.07(−0.24,0.38)
	CDMR	−0.02(−0.27,0.23)	−0.15(−0.41,0.11)	−0.19(−0.45,0.07)	0.01(−0.42,0.43)	0.26(−0.23,0.75)
	UCD	−0.17(−1.00.0.65)	0.07(−0.50,0.65)	−0.19(−0.76,0.38)	−0.71(−1.13,-0.29)*	−0.35(−1.70,1.00)
HO-1	CDMI	0.18(−0.28,0.64)	0.25(0.04,0.45)*	0.30(0.10,0.50)*	0.01(−0.25,0.26)	0.31(−0.09,0.71)
	CDMR	0.13(−0.17,0.43)	0.24(−0.04,0.52)	0.37(0.21,0.53)*	0.23(−0.01,0.48)	0.41(0.04,0.78)*
	UCD	0.11(−0.74,0.96)	0.26(−0.33,0.84)	−0.01(−0.31,0.28)	0.17(−0.68,1.02)	−0.18(−0.74,0.39)

Data were presented by quantile regression coefficients and 95% CIs (Confidence intervals) which based on above cytokines mRNA expression. Vaginal delivery group was regarded as control; *CDMI* cesarean delivery with medical indication, *CDMR* cesarean deliveryon maternal request, *UCD* urgent cesarean delivery

Model was adjusted by these variables: pre pregnancy BMI, maternal age, gravidity, previous adverse pregnant outcome, pregnancy-specific Anxiety Score in 1st, 2nd and 3rd trimester, birth weight, gestational age, infant gender and placental size

*P values less than 0.05 were considered significant (two-sided)

Delivery modes were associated with IL-1β, IL-6, IFN-γ, IL-8, IL-10 and HO-1 mRNA expression at different quintiles. At the 50th percentile of IL-1β, women with CDMI had significantly lower expression level than those with VD. Both at the 75th and 90th percentiles of IL-1β, the expression levels were lower in women with CDMR compared to those with VD. At the 10th percentile of IL-6, women with CDMI and CDMR had higher level compared with those with VD. At the 75th and 90th percentiles, the expression levels were higher in CDMR than VD. As for IL-8 mRNA expression, almost at all percentiles, the levels were lower in women with CDMR. At the 75th and 90th percentiles, the levels were lower in women with CDMI as well. At the 25th and 50th percentiles of HO-1 mRNA expression, women who chose CDMI had higher levels; while at the 50th and 90th percentiles, the levels were higher in women with CDMR.

In short, at median and high percentiles, women who had childbirth with elective caesarean delivery had lower expression of IL-1β and IFN-γ than women choosing vaginal delivery. In particular, women with CDMR had significantly lower level at high percentiles of IL-1β and IFN-γ. Women with CDMR had lower levels of IL-8 at low, median and high percentiles. At low and high percentiles of IL-6, women with CDMR had significantly higher level than women with VD. At median and high percentiles of HO-1, women with CDMR had higher level compared to those with VD. Women with CDMI had higher expression at low percentiles of IL-6, as well as at low and median percentiles of HO-1.

## Discussion

In current study, the overall rate of CDfound to be 50.5% in singleton live births in this study. Due to 112 missing data on reasons for choosing CD, we cannot calculate the accurate prevalence of ECD or UCD. Even so, we are concerned with the high CD rate observed here, which was higher than that reported by WHO multi-country surveys [3]. In addition, in women who reported definite reasons for CD, 56.8% chose the surgery without any medical indications. Caesarean delivery on maternal request has played the major role in the upward trend of CD rate. The reasons for the very highoverall cesarean delivery rate and CDMR in China are complex, social and cultural factors are the major contributors to the high cesarean delivery rate in China [27].

We have found a complex variation of cytokines related with placenta inflammatory response and oxidative stress response with different delivery modes, especially between elective caesarean delivery and vaginal delivery. It shows a non-linear relationship between these cytokines and delivery modes. In detail, at high percentiles of IL-1β, IFN-γ and IL-8, women with CDMR had higher expression levels compared to women with VD. Mothers with CDMI had higher levels at median percentiles of IL-1β, IFN-γ and IL-8. Women with CDMR had higher expression compared with VD at high percentiles of IL-6 and HO-1, and women with CDMI had higher levels of these two cytokines at their low percentiles. It is worth noting that at high percentiles, compared with normal delivery, the expression of IL-1β, IFN-γ, IL-8 and HO-1 have significantly altered in women with CDMR. The process of childbirth is accompanied by an increase in oxidative aggression.Delivering by CDMRs is not always less stressedthan those who deliver in a natural manner. It might be speculated that when the stress intensity is much strong, even without any medical conditions that can potentially affect the inflammatory and oxidative stress process, women will still suffer with obviously fluctuant levels of cytokines related to oxidative stress response and inflammatory response. Such endocrine alteration can cause negative outcomes in mothers and neonates. For example, a sudden increase in oxygenation exposes the neonate into oxidative stress since the DNA oxidative damage in mononuclear cells of umbilical blood as well as other indexes related to redox status [28]. The remarkable shift of oxidative stress and inflammatory response in surgery delivery on maternal request has provided the evidence that mothers and newborns from CDMRs may face more harmful potentials compared with their peers from natural spontaneous deliveries.

Our finding is partly consistent with the results reported by Keelan et al. [29], which in amniotic tissues of women delivered by spontaneous labor at term, the median IL-8, and IL-1β concentrations are 3.8 to 5.4 times those of tissues from women delivered at term without labor, while our findings on IL-6 are quite opposite to their results, as they still observe an elevated level in women with natural delivery. We have also obtained similar findings about IL-1β with the report by Gedikbaşi et al. [30], whose research team shows that cord blood IL-1β level of UCD group were significantly higher than those of ECD and normal delivery groups, and the level of IL-1β in women with normal delivery was significantly higher than those withECD. Bakheitet al [16] suggest that concentrations of IFN-γ, IL-4 and IL-10 in the placental sera were higher in vaginal delivery. However, we have not found significant difference in IL-4 among various delivery modes.

Gestation is a physiologic state in which oxygen demand is increased and high energy was required for various body functions. These increments in the in-taking and utilizing of oxygen lead to alterations in the oxidant-antioxidant balance, particularly toward the oxidant side, eventually causing oxidative stress and even damage. During parturition, this stress increases more profoundly [14]. Either vaginal delivery or cesarean section is a kind of stress to woman, which could lead to oxidative stress response and inflammatory response. Labor is associated with increased placental oxidative stress, and women with normal VD exhibit different oxidative stress indicators than those with ECD. Previous researchers found that VD is associated with increased placental oxidative stress compared to ECD [15]. However, Mutlu et al. [31] suggest that both the mothers and neonates in the CD group are exposed to higher oxidative stress as compared with those in the normal spontaneous vaginal deliveries group. During normal pregnancy, the influx of maternal blood flow in the placenta leads to a local increase of the production of reactive oxygen species (ROS). ROS induced oxidative stress can alter embryonic development and are also correlated with adverse pregnancy outcomes, such as miscarriage, preeclampsia, preterm labor, and fetal brain injury [12]. With the increase of ROS induced oxidative stress, oxidative stress proteins like HO-1 alter at the same time. In our study, as an antioxidant and anti-inflammatory biomarker [32], HO-1 mRNA was expressed higher in ECD group (including CDMI and CDMR) than was done in VD group, and this effect may be amplified at median and high percentiles of HO-1 in women with CDMR. At the same time, hypoxia and extracellular inflammatory signals induce the intracellular accumulation ofROS [33]. NF-κB resides in the cytoplasm in an inactive complex with the inhibitor I kappa B (IκB). ROS stimuli cause reduction of IκB for proteasomal degradation and allow NF-κB to enter the nucleus, bind to DNA control elements to induce gene expression. There is no doubt that CD after anesthesia can cause numerous release of ROS. In order to keep in the oxidant-antioxidant balance, the present results support the concept that HO-1, as an antioxidant could be part of the compensatory mechanism that HO-1mRNA expressed higher in ECD group.

Inflammation closely linked with oxidative stress and interacted on each other. Obviously, maternal oxidative stress may contribute towards placental inflammation [34]. The NF-κB pathway is an important bridge to link with each other. The NF-κB pathway has been implicated in responses to oxidative stress. Phosphorylation and subsequent degradation of the IκB protein allows translocation of NF-κB to the nucleus, where it regulates gene expression. The ratio of phosphorylated IκB to total protein increased evidently with the duration of labor, reflecting activation of the NF-κB pathway. Activation of NF-κB suggests an increase of pro-inflammatory cytokines after labor. A Previous study has confirmed that TNF-α, IL-1β were all significantly increased in labored placentas compared with cesarean controls [35]. IL-1β significantly depended on the mode of delivery. Our results showed the same trend in placental IL-1β mRNA expressionbeing lower in elective cesarean section, at the median and high percentiles. It specifically shows a significant decrease at high levels in women with CDMR, reflecting the amplified effect of CDMR in the condition of a stronger and more intensive stress. It is not clear that there is an opposite relationship between ECD and VD in IL-6 mRNA expression. Since there essentially exist complex interactions among cytokines. In the class of pro-inflammatory cytokines, there may be an overall increasing trend in vaginal delivery compared with ECD but each cytokine may play its own role and thus not all pro-inflammatory cytokines express an uprising level.

As one of pro-inflammatory chemokines, IL-8 is the prototype of some chemokines, attracts polymorphonuclear leukocytes to sites of acute inflammation. IL-8 also activatesmonocytes [26]. We observed that IL-8 mRNA expression attenuate in CDMI and CDMR compared with VD almost at all percentiles. IL-1β is mainly produced by monocytes and macrophages [11, 36], and thus, there are significant associations between pro-inflammatory chemokines (like IL-8) and pro-inflammatory cytokines (such as IL-1β).

Our research has some strengths. Firstly, our study samples were much larger than previous studies, and would likely increase the statistical validity. Secondly, by using a longitudinal study design, pregnancy-related information was collected with appropriate physical examination and questionnaire surveys in all trimesters of pregnancy, allowing well control for multiple confounding variables, e.g., pregnancy-specific anxiety during the three trimesters of pregnancy and common pregnant complications. Thirdly, as there is a non-linear relationship between delivery modes and oxidative stress and inflammatory response, we have adopted quintile regression model to divide the cytokines expression into low (10th, 25th), median (50th) and high (75th, 90th) level. It has specific advantages to show the actual association at different concentration levels where this subtle relevance can’t be displayed in traditional linear regression models.

Several limitations of this study should also be noted.Although we were able to control for some prenatal confounding factors, we could not rule out the potential for residual confounding as it is impossible to fully account for all potential factors that may be associated with placental cytokines mRNA expression and delivery mode. Meanwhile, not all inflammatory and oxidative stress cytokines were examined in this study, and only representative cytokines were selected.

## Conclusion

In conclusion, we have reported a high overall CD rate in this study, and caesarean delivery on maternal request is the major contributor to the high prevalence, which supports previous findings reported by Zhang et al. [5]. Maternal placental IL-1β, IL-6, IL-8 and HO-1 mRNA expressions were closely associated with delivery mode in this study, with significant differences between elective caesarean deliveryand natural delivery. The effect is much amplified at high levels of expression in women who chose CD on maternal request. Under the condition of high CD rate, especially non medically-indicated ECD contributed mainly to the high level, such difference needs to be noticed and may have important implications on obstetricians, midwives and other perinatal health care workers. Inflammatory and oxidative stress is a complex network in which every cytokine plays its own role and interacts with each other, and may influence mothers’ and infants’ health. Further research is warranted to explain the mechanism of various cytokines’ alteration patterns in different delivery modes. The effect on maternal and child health of different levels of oxidative stress and inflammatory cytokines is worth investigating and it is important to assess the short-term and long-term health outcomes caused by these delivery-mode-dependent biomarkers.

## Abbreviations

BMI: Body mass index
CDMI: Cesarean delivery with medical indication
CDMR: Cesarean deliveryon maternal request
CI: Confidence interval
ECD: Elective caesarean delivery
GDM: Gestational diabetes mellitus
HDCP: Hypertension disorders complicating pregnancy
ICP: Intrahepatic cholestasis of pregnancy
MBCS: Ma’anshan Birth Cohort Study
SD: Standard deviation
SE: Standard error
UCD: Urgent cesarean delivery
VD: Vaginal delivery
